# Targeting MicroRNA-125b Promotes Neurite Outgrowth but Represses Cell Apoptosis and Inflammation via Blocking PTGS2 and CDK5 in a FOXQ1-Dependent Way in Alzheimer Disease

**DOI:** 10.3389/fncel.2020.587747

**Published:** 2020-12-21

**Authors:** Jingcong Zhuang, Zhongjie Chen, Pingping Cai, Rong Wang, Qingwei Yang, Longling Li, Huili Yang, Renjing Zhu

**Affiliations:** ^1^Department of Neurology, Zhongshan Hospital Xiamen University, Xiamen, China; ^2^School of Medicine, Xiamen University, Xiamen, China; ^3^Department of Neurology, Fujian Medical University Xiamen Humanity Hospital, Xiamen, China

**Keywords:** MicroRNA-125b, Alzheimer's disease, FOXQ1, PTGS2, CDK5

## Abstract

This study aimed to explore the molecular regulatory network among microRNA-125b (miR-125b), forkhead box Q1 (FOXQ1), prostaglandin-endoperoxide synthase 2 (PTGS2), and cyclin-dependent kinase 5 (CDK5), as well as their effects on cell apoptosis, neurite outgrowth, and inflammation in Alzheimer disease (AD). Rat embryo cerebral cortex neurons and nerve growth factor–stimulated PC12 cells were insulted by Aβ_1−42_ to construct two AD cellular models. Negative control (NC) inhibitor, miR-125b inhibitor, NC siRNA, FOXQ1 siRNA, PTGS2 siRNA, and CDK5 siRNA were transferred into the two AD cellular models alone or combined. Then, cell apoptosis, neurite outgrowth, proinflammatory cytokines, miR-125b, FOXQ1, PTGS2, and CDK5 expressions were detected. MiR-125b inhibition facilitated neurite outgrowth but suppressed cell apoptosis and proinflammatory cytokines (tumor necrosis factor-α, interleukin 1β, and interleukin 6); meanwhile, it upregulated FOXQ1 but downregulated PTGS2 and CDK5. Furthermore, FOXQ1 inhibition promoted cell apoptosis and proinflammatory cytokines but repressed neurite outgrowth; PTGS2 inhibition achieved the opposite effects; CDK5 inhibition attenuated cell apoptosis, whereas it less affected neurite outgrowth and inflammation. Notably, FOXQ1 inhibition attenuated, whereas PTGS2 inhibition elevated the effect of miR-125b inhibition on regulating neurite outgrowth, cell apoptosis, and proinflammatory cytokines. As for CDK5 inhibition, it enhanced the effect of miR-125b inhibition on regulating cell apoptosis, but less impacted the neurite outgrowth and proinflammatory cytokines. Additionally, PTGS2 inhibition and CDK5 inhibition both reversed the effect of FOXQ1 inhibition on regulating cell apoptosis, neurite outgrowth, and proinflammatory cytokines. In conclusion, targeting miR-125b alleviates AD progression via blocking PTGS2 and CDK5 in a FOXQ1-dependent way.

## Introduction

Alzheimer disease (AD) represents a devastating, age-related neurodegenerative disease with a long asymptomatic preclinical phase (more than 20 years) and an average clinical duration of 10 years, affecting ~35 million individuals worldwide (Masters et al., 2015; Liu et al., 2019). It is characterized by major pathological hallmarks in the brain including the deposition of extracellular amyloid-β plaques and intracellular neurofibrillary tangles (Gjoneska et al., 2015). The progressive accumulation of amyloid-β plaques and neurofibrillary tangles is related to synapse loss and neurodegeneration in hippocampus (involved in memory) and cerebral cortex (involved in language, reasoning, and social behaviors), which drives irreversible progressive deterioration of memory, cognitive decline, impaired language skill, and personality changes (Scheltens et al., 2016; Liu et al., 2019). Although a large amount of research has been dedicated to understanding the basic biology and clinical pathophysiology underlying AD, current treatments are limited to few drugs with short-term symptomatic efficacy (Liu et al., 2019). Therefore, the elucidation of the basic disease pathophysiology underlying AD might pave the way for developing innovative drug targets to combat this disease.

MicroRNA-125b (MiR-125b), one of the most abundant miRNAs in the brain, is closely linked to the regulation of tau phosphorylation, neuroinflammation, and neuron apoptosis by the modulation of inflammatory factors and oxidative stress, which is implicated in the pathogenesis of AD (Banzhaf-Strathmann et al., 2014; Graham et al., 2017; Jin et al., 2018; Ma et al., 2019). For instance, Banzhaf-Strathmann et al. (2014) reveal that the overexpression of miR-125b induces the tau hyperphosphorylation through the activation of p35, cdk5, p44/42–mitogen-activated protein kinase signaling and the downregulation of dual specificity phosphatase 6 and protein phosphatase 1 catalytic subunit alpha in both *in vitro* and *in vivo*. In the study of Jin et al. based on an *in vitro* model of AD, overexpression of miR-125b suppressed neuron proliferation but enhanced its apoptosis, inflammation, and oxidative stress (Jin et al., 2018). In addition, one previous study conducted by our collaborate institution discloses that miR-125b participates in regulating the pathogenesis of AD, forkhead box Q1 (FOXQ1), prostaglandin-endoperoxide synthase 2 (PTGS2), and cyclin-dependent kinase 5 (CDK5) expressions in AD cellular models (Ma et al., 2019). Meanwhile, miR-125b is also reported to induce neuronal cell apoptosis, tau phosphorylation, and target FOXQ1 in AD cells (Ma et al., 2017). However, the molecular regulatory network among miR-125b, FOXQ1, PTGS2, and CDK5, as well as their regulatory effects on the functions of AD cellular models, is not evaluated yet.

In this current study, we aimed to investigate the molecular regulatory network among miR-125b, FOXQ1, PTGS2, and CDK5, as well as their effects on cell apoptosis, neurite outgrowth, and inflammation in AD.

## Methods

### Primary Cerebral Cortex Neurons and PC-12 Cell Culture

Two pregnant rats were used, and the primary cerebral cortex neurons were isolated from Sprague–Dawley rat embryo carried by pregnant rats according to the methods described in a previous study (Yang et al., 2018). After the isolation, the cells were cultured in neurobasal medium (Gibco, USA) supplemented with 2% B-27 (Gibco, USA). PC-12 cells (ATCC, USA) were cultured in RPMI-1640 medium supplemented with 10% horse serum (Gibco, USA) and 5% fetal bovine serum (FBS) (Gibco, USA). All cells were cultured in a humidified atmosphere of 5% CO_2_ at 37°C. For the differentiation induction, PC-12 cells were cultured in RPMI-1640 medium containing 100 ng/mL nerve growth factor (NGF) (Sigma, USA) and 10% FBS (Gibco, USA) for 72 h. The animal-related experiment was completed in accordance with the National Guideline for Experimental Animal Welfare and approved by the Animal Ethics Committee of our institution. Each experiment was conducted in biological triplicates (*n* = 3) and a single technical replicate (*n* = 1).

### Aβ_1−42_ Insult

Aβ_1−42_ (Sigma, USA) dissolved in dimethyl sulfoxide at 10 mM was used to induce aggregation at 37°C for 7 days. Different concentrations of aggregated Aβ_1−42_ (0, 0.1, 1, 10, and 20 μM) in culture medium were cultured with primary cerebral cortex neurons and NGF-stimulated PC-12 cells for 24 h. Cell viability was determined by cell counting kit-8 (Dojindo, Japan) following the instruction, and the expression of miR-125b was evaluated by reverse transcription–quantitative polymerase chain reaction (RT-qPCR). According to the relative cell viability under differed concentration of Aβ_1−42_, 10 μM aggregated Aβ_1−42_-treated primary cerebral cortex neurons and NGF-stimulated PC-12 cells were identified as cellular AD models. Meanwhile, as for primary cerebral cortex neurons and NGF-stimulated PC-12 cells without Aβ_1−42_ treatment, they were identified as normal cells. Furthermore, in both cellular AD models and normal cells, FOXQ1, PTGS2, CDK5 expressions, cell apoptosis, neurite outgrowth, and inflammation were detected.

### Transfection

After Aβ_1−42_ treatment, transfection was performed. MiR-125b inhibitor (5′-UCACAAGUUAGGGUCUCAGGGA-3′; Genepharma, China) and negative control (NC) inhibitor (5′-CAGUACUUUUGUGUAGUACAA-3′; Genepharma, China) were transfected into primary neuron AD model and PC-12 cellular AD model (Aβ_1−42_-treated primary neurons and Aβ_1−42_-treated NGF-stimulated PC-12 cells) with Lipofectamine™ LTX Reagent with PLUS™ Reagent (Invitrogen, USA). The cells transfected with miR-125b inhibitor (Genepharma, China) and NC inhibitor (Genepharma, China) were named as inhibitor-miR group and NC group, respectively, and the cells without transfection were named as blank group. Besides, FOXQ1 siRNA (5′-CGCGGACUUUGCACUUUGA-3′; Genepharma, China) was transfected into normal cells with the application of Lipofectamine™ LTX Reagent with PLUS™ Reagent (Invitrogen, USA); the cells transfected with FOXQ1 siRNA (Genepharma, China) were named as Si-FOXQ1 group. At 24 h after transfection, the expression of miR-125b was determined by RT-qPCR. At 48 h, the cell apoptosis rate was evaluated by Hoechst/propidium iodide (PI) (Sigma, USA) according to the methods in the previous study (Ma et al., 2019), and the cell apoptosis rate was measured by ImageJ. Five-field views (under ×100 magnification) were analyzed. In each field view (under ×100 magnification), cell apoptosis rate was calculated via the number of apoptotic cells divided by the total number of cells and then displayed as a percentage. Subsequently, the mean value of cell apoptosis rate of five field views was taken as the result. Neurite outgrowth was observed under ×200 magnification with a microscope (Olympus, Japan), and the neurite outgrowth was measured by ImageJ. Five-field views (under ×200 magnification) were assessed. In each field view (under ×200 magnification), neurite outgrowth was calculated via the total neurite length outgrowth divided by the total number of cells. Then, the mean value of neurite outgrowth of five-field views was taken as the result. Besides, the TNF-α, interleukin 1β (IL-1β), and IL-6 in supernatant were measured by enzyme-linked immunosorbent assay (ELISA), and the expressions of FOXQ1, PTGS2, and CDK5 in each group were detected by Western blot.

### MiR-125b Rescue Experiment

At the preliminary stage of our experiments, three kinds of siRNAs were generated for FOXQ1, PTGS2, or CDK5 respectively, and then the siRNA with best knockdown efficiency was selected for the cellular experiments. NC inhibitor (Genepharma, China), miR-125b inhibitor (Genepharma, China), FOXQ1 siRNA (Genepharma, China), miR-125b Inhibitor (Genepharma, China) combining FOXQ1 siRNA (Genepharma, China), PTGS2 siRNA (5′-UCAAGUGUUGCACAUAAUCDTDT-3′; Genepharma, China), miR-125b inhibitor (Genepharma, China) combining PTGS2 siRNA (Genepharma, China), CDK5 siRNA (5′-GUCGAUGACCAGUUGAAGATT-3′; Genepharma, China), and miR-125b inhibitor (Genepharma, China) combining CDK5 siRNA (Genepharma, China) were transfected into primary neuron AD model and PC-12 cellular AD model (Aβ_1−42_-treated primary neurons and Aβ_1−42_-treated NGF-stimulated PC-12 cells) with the application of Lipofectamine™ LTX Reagent with PLUS™ Reagent (Invitrogen, USA). And the transfected cells were termed as NC group, inhibitor-miR group, Si-FOXQ1 group, inhibitor-miR&Si-FOXQ1 group, Si-PTGS2 group, inhibitor-miR&Si-PTGS2 group, Si-CDK5 group, and inhibitor-miR&Si-CDK5 group, accordingly. At 48 h, the cell apoptosis rate was evaluated by Hoechst/PI (Sigma, USA) following the method described in the previous study (Ma et al., 2019); neurite outgrowth was observed under ×200 magnification with a microscope (Olympus, Japan), and then the TNF-α, IL-1β, and IL-6 in supernatant were measured by ELISA, and the expressions of FOXQ1, PTGS2, and CDK5 in each group were detected by Western blot.

### FOXQ1 Rescue Experiment

NC siRNA (5′-UUCUCCGAACGUGUCACGU-3′; Genepharma, China), FOXQ1 siRNA (5′-CGCGGACUUUGCACUUUGA-3′; Genepharma, China), PTGS2 siRNA (5′-UCAAGUGUUGCACAUAAUCDTDT-3′; Genepharma, China), FOXQ1 siRNA (5′-CGCGGACUUUGCACUUUGA-3′; Genepharma, China) combining PTGS2 siRNA (5′-UCAAGUGUUGCACAUAAUCDTDT-3′; Genepharma, China), CDK5 siRNA (5′-GUCGAUGACCAGUUGAAGATT-3′; Genepharma, China), and FOXQ1 siRNA (5′-CGCGGACUUUGCACUUUGA-3′; Genepharma, China) combining CDK5 siRNA (5′-GUCGAUGACCAGUUGAAGATT-3′; Genepharma, China) were transfected into two cellular AD models (primary neuron AD model and PC-12 cellular AD model) using Lipofectamine™ LTX Reagent with PLUS™ Reagent (Invitrogen, USA). Then the transfected cells were divided into NC group, Si-FOXQ1 group, Si-PTGS2 group, Si-FOXQ1&Si-PTGS2 group, Si-CDK5 group, and Si-FOXQ1&Si-CDK5 group. At 48 h, the cell apoptosis rate was evaluated by Hoechst/PI (Sigma, USA) in accordance with the method in the previous study (Ma et al., 2019); neurite outgrowth was observed under ×200 magnification with a microscope (Olympus, Japan), and then the TNF-α, IL-1β, and IL-6 in supernatant were measured by ELISA, and the expressions of FOXQ1, PTGS2, and CDK5 in each group were detected by Western blot.

### Reverse Transcription–Quantitative Polymerase Chain Reaction

Total RNA was isolated using TRIzol Reagent (Invitrogen, USA). After that, total RNA was reversely transcribed to complementary DNA using PrimeScript™ RT Reagent Kit (Takara, Japan). The following qPCR procedure was conducted using SYBR® Premix DimerEraser™ (Takara, Japan). Lastly, qPCR results were calculated by the 2^−ΔΔCt^ method with U6 as an internal reference. The primer sequences applied were as follows: miR-125b, forward, 5′-ACACTCCAGCTGGGTCCCTGAGACCCTAACTT-3′, reverse, 5′-TGTCGTGGAGTCGGCAATTC-3′; U6, forward, 5′-CGCTTCGGCAGCACATATACTA-3′, reverse, 5′-ATGGAACGCTTCACGAATTTGC-3′.

### Western Blot

After the collection of cells, the total protein was extracted by RIPA Lysis Buffer (Sigma, USA), and then Pierce™ BCA Protein Assay Kit (Thermo, USA) was used for protein quantification. After denaturation, the protein was separated on TruPAGE™ Precast Gels (Sigma, USA). Subsequently, the protein was transferred to nitrocellulose membrane (Millipore, USA). Membrane was incubated with the primary antibodies at 4°C overnight. Following that, membrane was incubated with secondary antibody for 1 h at room temperature. Lastly, the protein was visualized by ECL Plus Western Blotting Substrate (Pierce, USA). The antibodies used in Western blot were as follows: primary antibodies, rabbit polyclonal to FOXQ1 (1:500 dilution, Abcam, UK), rabbit monoclonal to PTGS2 (1:2,000 dilution, Abcam, UK), rabbit monoclonal to CDK5 (1:5,000 dilution, Abcam, UK), rabbit monoclonal to GAPDH (1:10,000 dilution, Abcam, UK), secondary antibody, and goat anti–rabbit immunoglobulin G H&L (HRP) (1:5,000 dilution, Abcam, UK).

### Enzyme-Linked Immunosorbent Assay

The TNF-α Rat ELISA Kit (Invitrogen, USA), IL-1β Rat ELISA Kit (Invitrogen, USA), and IL-6 Rat ELISA Kit (Invitrogen, USA) were used to measure the TNF-α, IL-1β, and IL-6 in supernatant in accordance with the kits' instructions.

### Statistical Analysis

Statistical analysis and graph plotting were performed with GraphPad Prism Software version 7.0 (GraphPad Software Inc., USA). Data in this study are presented as mean and standard deviation. Comparisons among group were determined by one-way analysis of variance followed by Dunnett multiple-comparisons test or Tukey multiple-comparisons test. *P* < 0.05 was considered as statistically significant. ^*^, ^**^, and ^***^ represented *P* < 0.05, *P* < 0.01, and *P* < 0.001, respectively. *P* > 0.05 represents nonsignificance (NS).

## Results

### Cell Viability and miR-125b Expression After Aβ_1−42_ Insult Treatment

**Figure 1:**
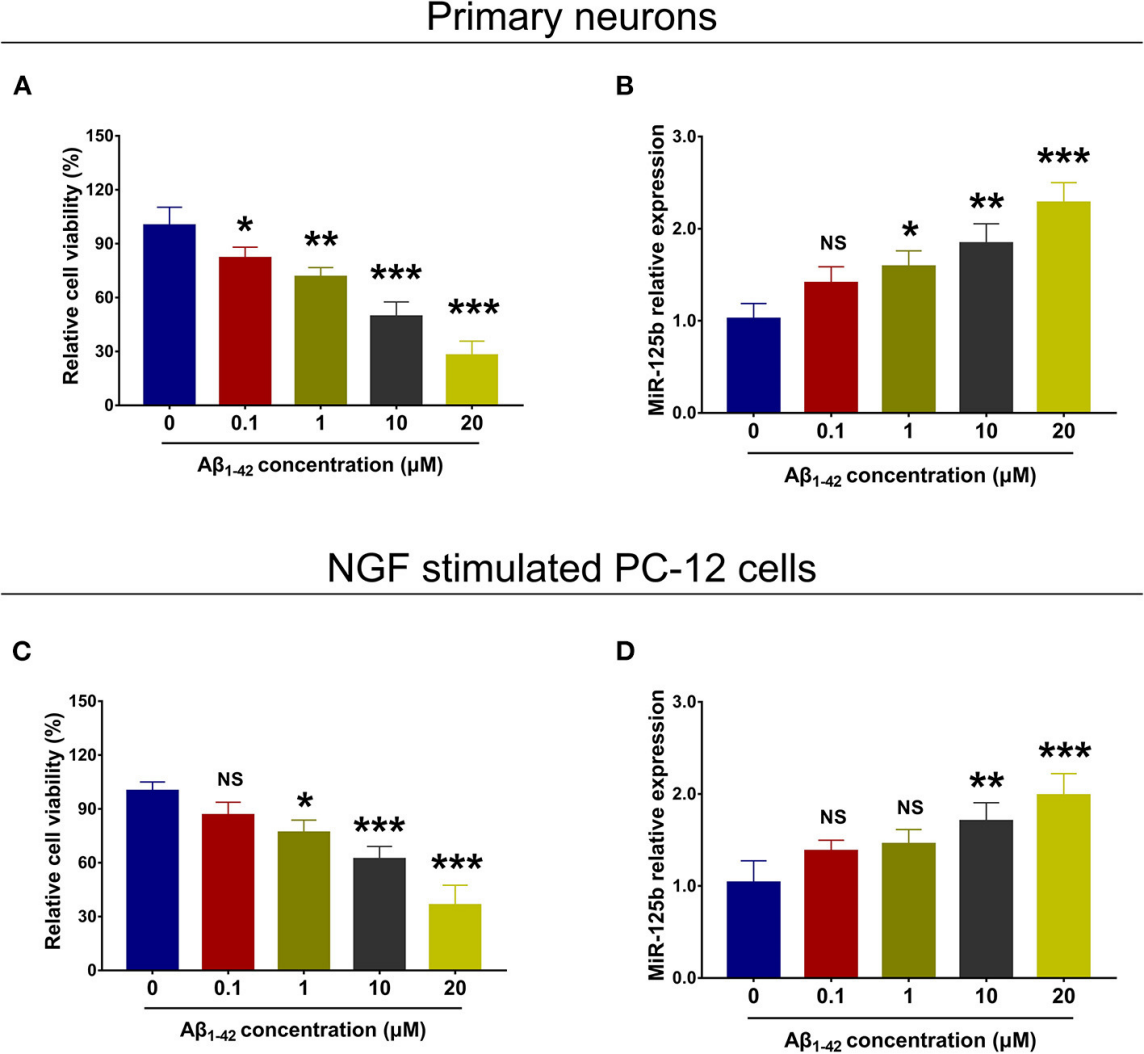
Cell viability and miR-125b relative expression in Aβ_1−42_-treated primary neurons and NGF-stimulated PC-12 cells. Comparisons of relative cell viability **(A)** and miR-125b relative expression **(B)** among primary neurons under the treatment of different Aβ_1−42_ concentrations (0, 0.1, 1, 10, and 20 μM). Comparisons of relative cell viability **(C)** and miR-125b relative expression **(D)** among NGF-stimulated PC-12 cells under the treatment of different Aβ_1−42_ concentrations (0, 0.1, 1, 10, and 20 μM). MiR-125b, microRNA-125b; NGF, nerve growth factor. *p < 0.05, **p < 0.01, ***p < 0.001.

In both primary neurons (Figures 1A,B) and NGF-stimulated PC-12 cells (Figures 1C,D), relative cell viability was decreased with increasing concentration of Aβ_1−42_, whereas miR-125b relative expression was elevated with increasing concentration of Aβ_1−42_.

### Aβ_1−42_ Treatment Promoted FOXQ1, PTGS2, CDK5, Cell Apoptosis, and Inflammation but Inhibited Neurite Outgrowth

The effect of Aβ_1−42_ treatment on FOXQ1, PTGS2, and CDK5 expression and cell activities was evaluated. In both primary neurons (Supplementary Figures 1A–F, 2A–C) and NGF-stimulated PC-12 cells (Supplementary Figures 1G–L, 2D–F), FOXQ1 expression and neurite outgrowth were decreased, while PTGS2 and CDK5 expressions, cell apoptosis, and inflammation (including TNF-α, IL-1β, and IL-6) were increased in AD models compared with normal cells.

### MiR-125b Inhibition Decreased Cell Apoptosis and Inflammation but Increased Neurite Outgrowth

**Figure 2:**
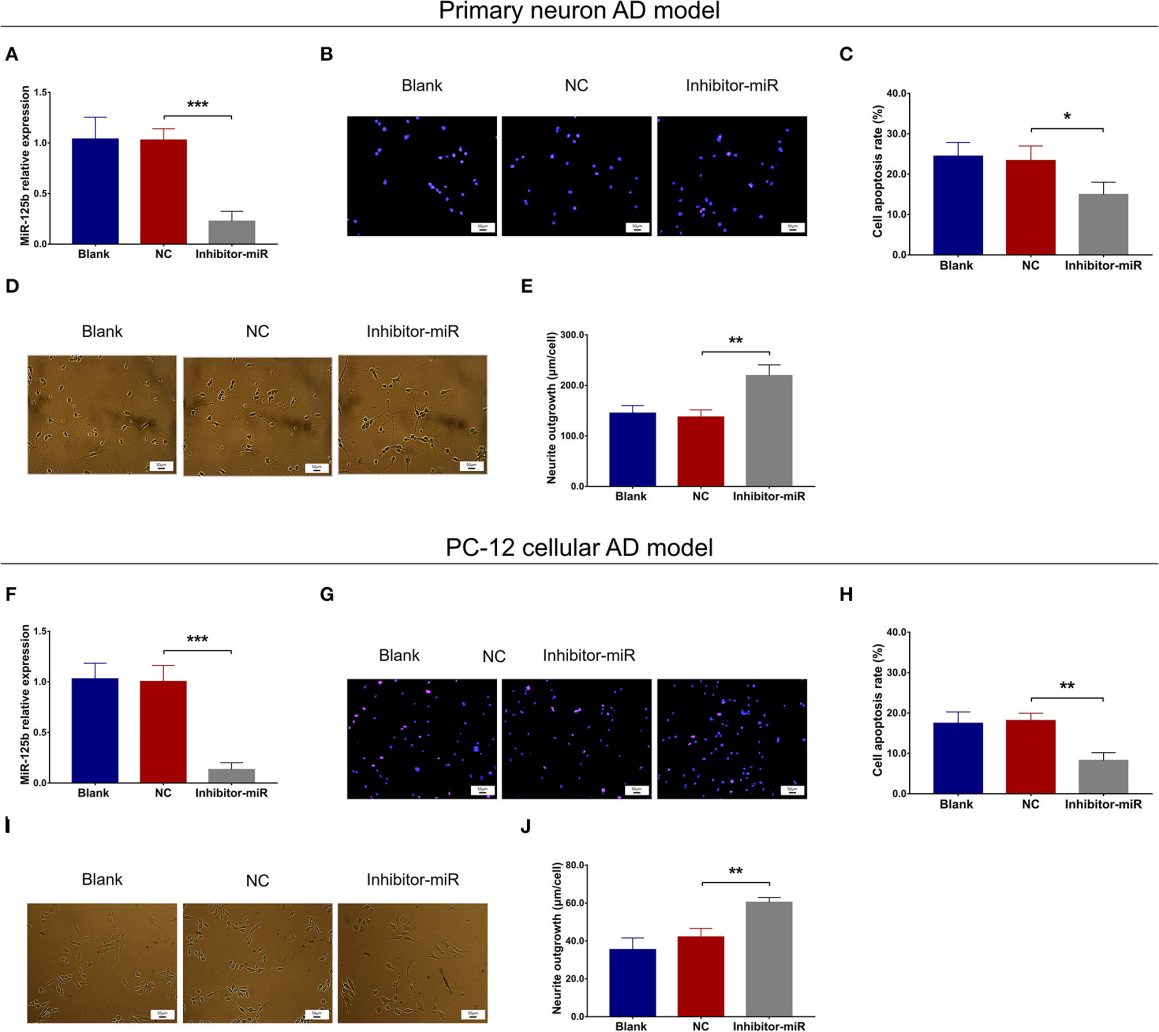
Effect of miR-125b inhibition on cell apoptosis and neurite outgrowth. Comparisons of miR-125b relative expression **(A)**, cell apoptosis **(B,C)**, and neurite outgrowth **(D,E)** between inhibitor-miR cells and NC cells in primary neuron AD model. Comparisons of miR-125b relative expression **(F)**, cell apoptosis **(G,H)**, and neurite outgrowth **(I,J)** between inhibitor-miR cells and NC cells in PC-12 cellular AD model. MiR-125b, microRNA-125b; AD, Alzheimer disease; NC, negative control. *p < 0.05, **p < 0.01, ***p < 0.001.

**Figure 3:**
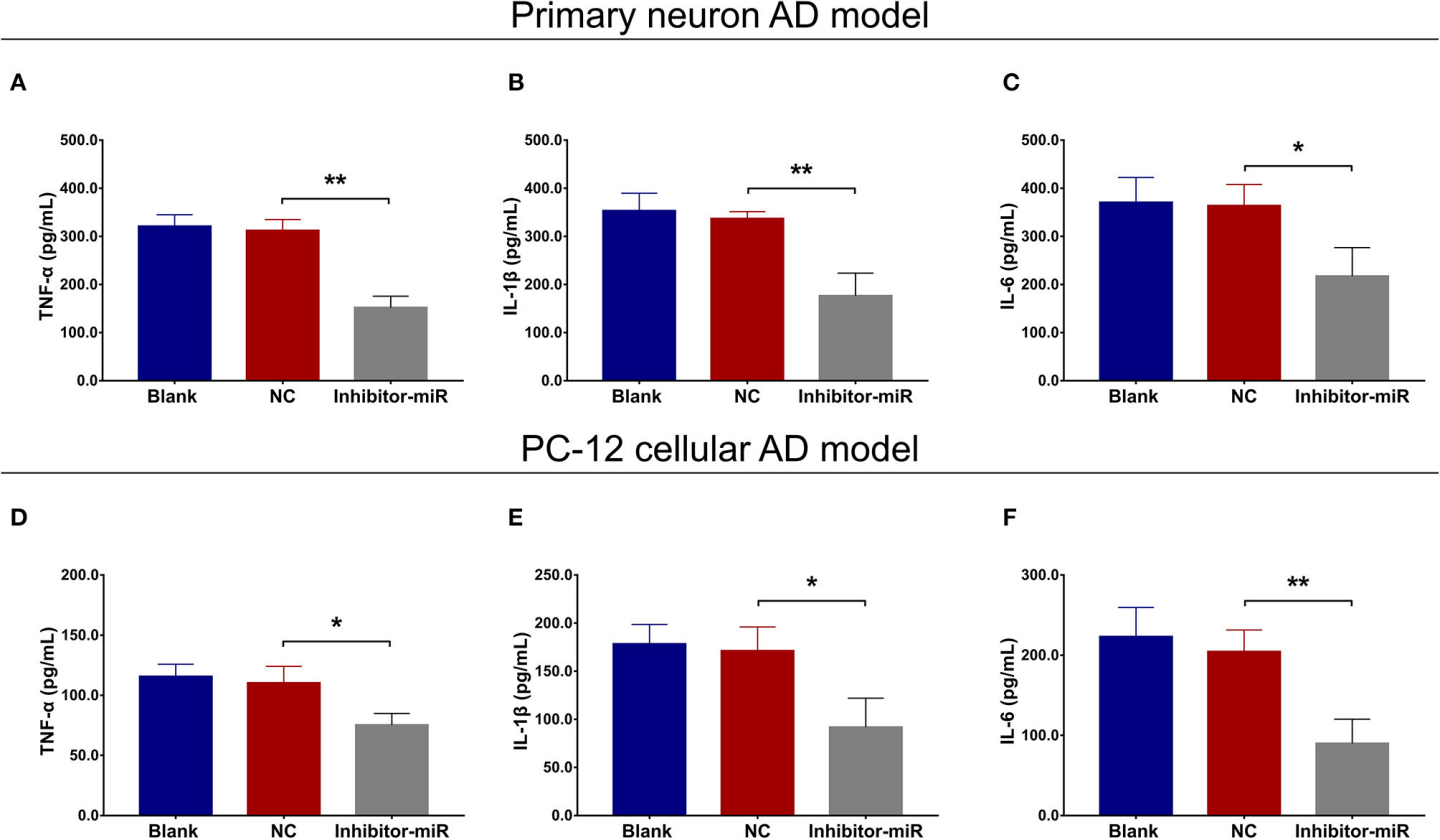
Effect of miR-125b inhibition on inflammation. Comparisons of TNF-α **(A)**, IL-1β **(B)**, and IL-6 **(C)** levels between inhibitor-miR cells and NC cells in primary neuron AD model. Comparisons of TNF-α **(D)**, IL-1β **(E)**, and IL-6 **(F)** levels between inhibitor-miR cells and NC cells in PC-12 cellular AD model. TNF-α, tumor necrosis factor α; IL-1β, interleukin 1β; IL-6, interleukin 6; AD, Alzheimer disease; NC, negative control. *p < 0.05, **p < 0.01.

In both primary neuron AD model (Figures 2A–E) and PC-12 cellular AD model (Figures 2F–J), miR-125b relative expression and cell apoptosis were reduced, whereas neurite outgrowth was increased in inhibitor-miR cells compared with NC cells. As for inflammation, in both primary neuron AD model (Figures 3A–C) and PC-12 cellular AD model (Figures 3D–F), TNF-α, IL-1β, and IL-6 levels were all attenuated in inhibitor-miR cells compared with NC cells. Collectively, these findings indicated that miR-125b inhibition suppressed cell apoptosis and inflammation but facilitated neurite outgrowth in AD.

### MiR-125b Inhibition Promoted FOXQ1 Expression but Impeded PTGS2 and CDK5 Expressions

**Figure 4:**
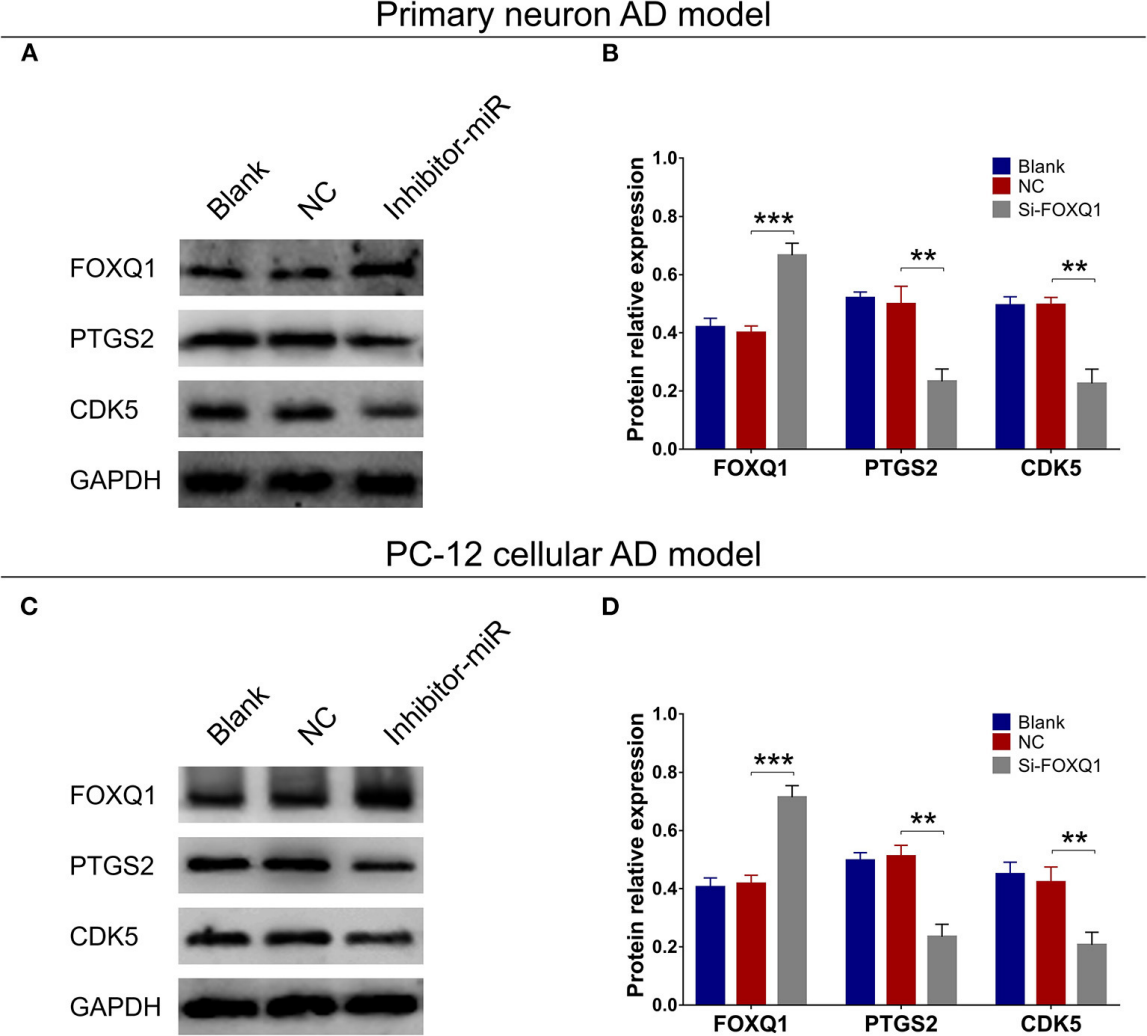
Effect of miR-125b inhibition on FOXQ1, PTGS2, and CDK5 expressions. Comparisons of FOXQ1, PTGS2, and CDK5 expressions **(A,B)** between inhibitor-miR cells and NC cells in primary neuron AD model. Comparisons of FOXQ1, PTGS2, and CDK5 expressions **(C,D)** between inhibitor-miR cells and NC cells in PC-12 cellular AD model. FOXQ1, forkhead box Q1; PTGS2, prostaglandin-endoperoxide synthase 2; CDK5, cyclin-dependent kinase 5; AD, Alzheimer disease; NC, negative control. **p < 0.01, ***p < 0.001.

In both primary neuron AD model (Figures 4A,B) and PC-12 cellular AD model (Figures 4C,D), FOXQ1 expression was increased, whereas PTGS2 and CDK5 expressions were reduced in inhibitor-miR cells compared with NC cells.

### MiR-125b Inhibition Repressed Cell Apoptosis and Inflammation but Enhanced Neurite Outgrowth by Upregulating FOXQ1

**Figure 5:**
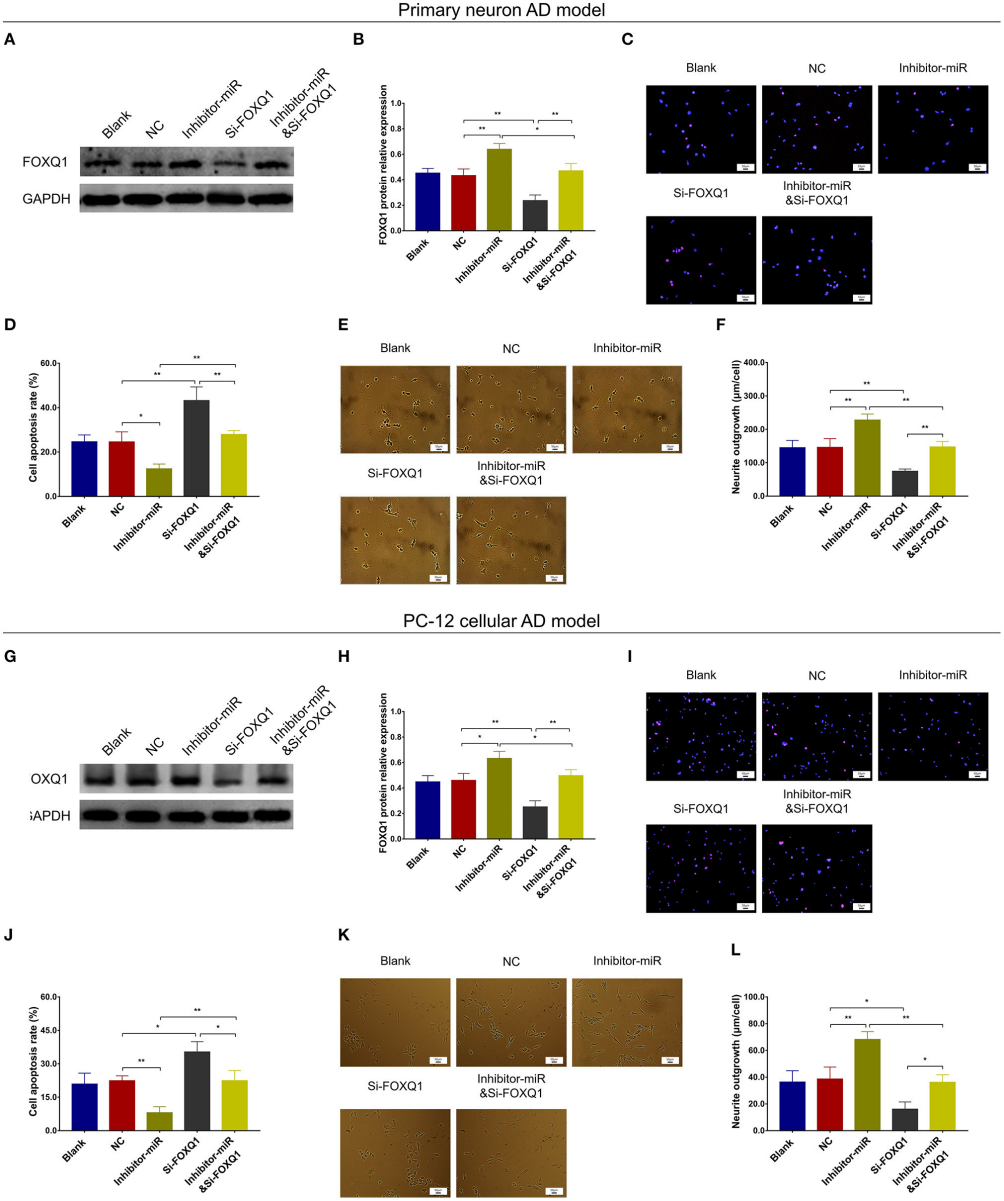
FOXQ1 inhibition compensated the effect of miR-125b inhibition on cell apoptosis and neurite outgrowth. Comparisons of FOXQ1 expression **(A,B)**, cell apoptosis **(C,D)**, and neurite outgrowth **(E,F)** among blank, NC, inhibitor-miR, Si-FOXQ1, and inhibitor-miRandSi-FOXQ1 cells in primary neuron AD model. Comparisons of FOXQ1 expression **(G,H)**, cell apoptosis **(I,J)**, and neurite outgrowth **(K,L)** among blank, NC, inhibitor-miR, Si-FOXQ1, and inhibitor-miRandSi-FOXQ1 cells in PC-12 cellular AD model. MiR-125b, microRNA-125b; FOXQ1, forkhead box Q1; AD, Alzheimer disease; NC, negative control. *p < 0.05, **p < 0.01.

**Figure 6:**
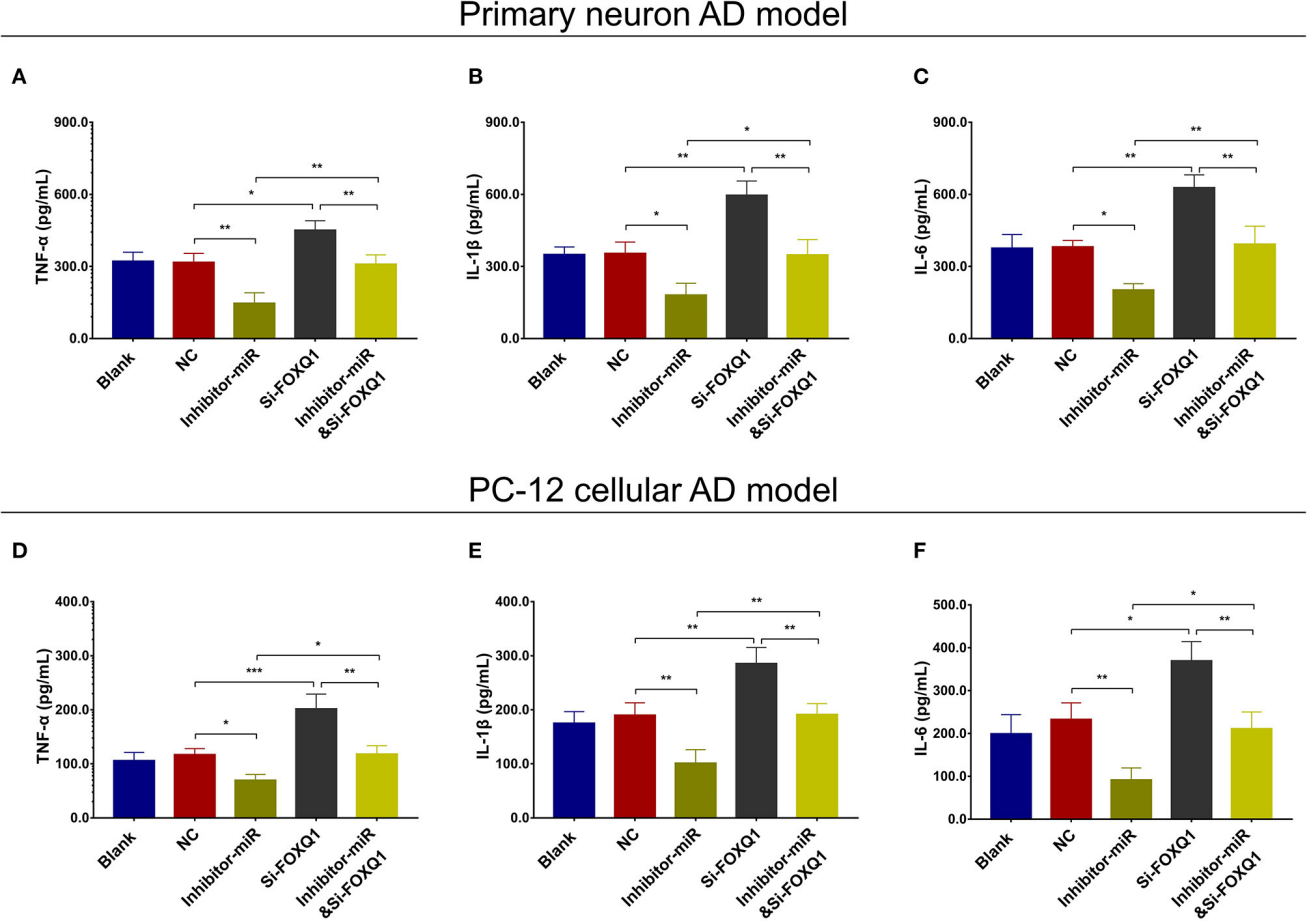
FOXQ1 inhibition compensated the effect of miR-125 inhibition on inflammation. Comparisons of TNF-α **(A)**, IL-1β **(B)**, and IL-6 **(C)** levels among blank, NC, inhibitor-miR, Si-FOXQ1, and inhibitor-miRandSi-FOXQ1 cells in primary neuron AD model. Comparisons of TNF-α **(D)**, IL-1β **(E)**, and IL-6 **(F)** levels among blank, NC, inhibitor-miR, Si-FOXQ1, and inhibitor-miRandSi-FOXQ1 cells in PC-12 cellular AD model. MiR-125b, microRNA-125b; FOXQ1, forkhead box Q1; TNF-α, tumor necrosis factor-α; IL-1β, interleukin 1β; IL-6, interleukin 6; NC, negative control; AD, Alzheimer disease. *p < 0.05, **p < 0.01, ***p < 0.001.

In NC or miR-125b inhibitor–treated primary neuron AD model (Figures 5A–F), as well as in NC or miR-125b inhibitor–treated PC-12 cellular AD model (Figures 5G–L), Si-FOXQ1 decreased FOXQ1 expression and neurite outgrowth and increased cell apoptosis, while Si-FOXQ1 did not affect miR-125b expression (Figures 5A–L, Supplementary Figure 3); meanwhile, Si-FOXQ1 also upregulated TNF-α, IL-1β, and IL-6 levels (Figures 6A–F). Collectively, these findings indicated that miR-125b inhibition suppressed cell apoptosis and inflammation but enhanced neurite outgrowth via upregulating FOXQ1 expression in AD.

### MiR-125b Inhibition Suppressed Cell Apoptosis and Inflammation but Enhanced Neurite Outgrowth by Downregulating PTGS2

**Figure 7:**
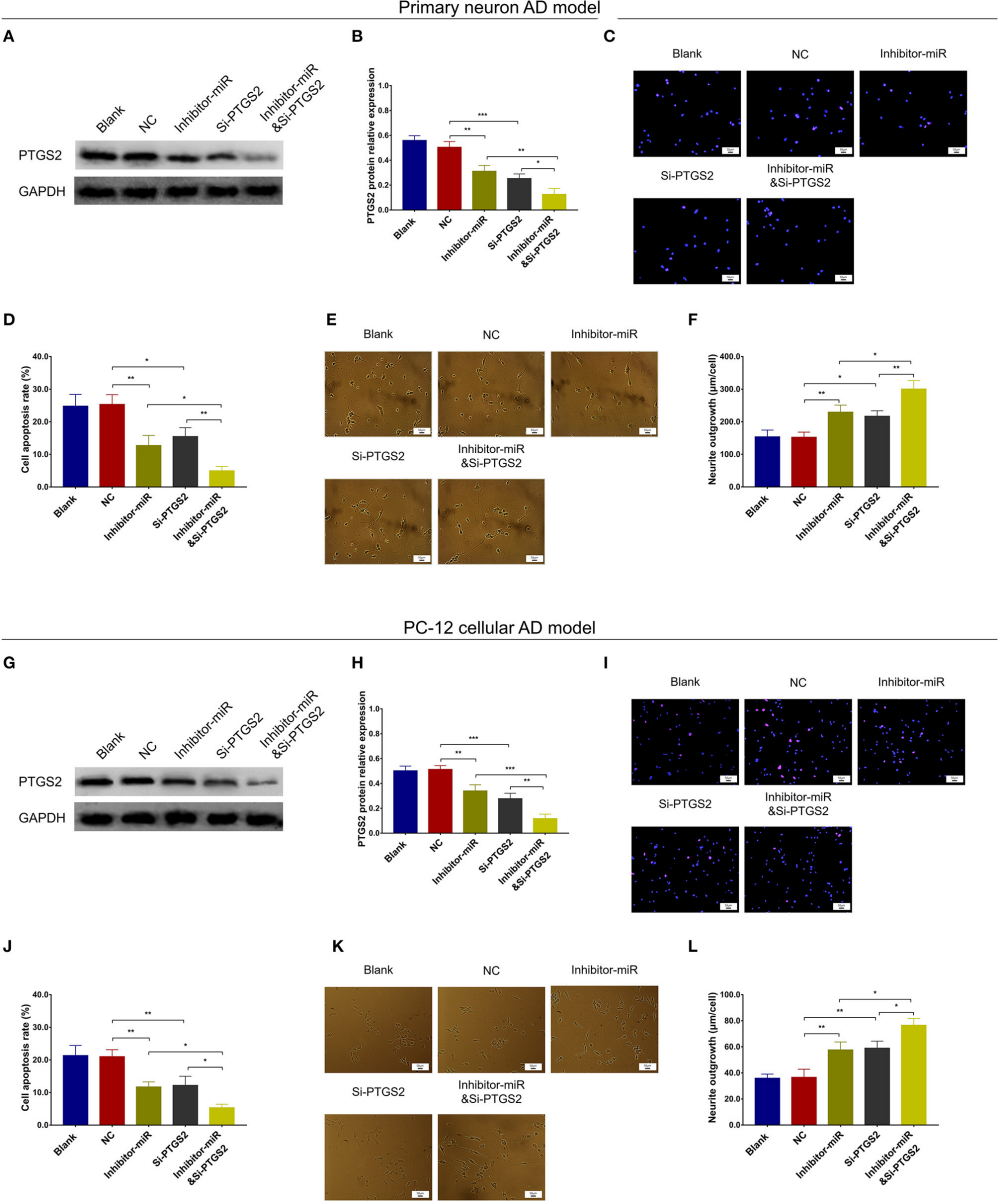
PTGS2 inhibition enhanced the effect of miR-125b inhibition on cell apoptosis and neurite outgrowth. Comparisons of PTGS2 expression **(A,B)**, cell apoptosis **(C,D)**, and neurite outgrowth **(E,F)** among blank, NC, inhibitor-miR, Si-PTGS2, and inhibitor-miRandSi-PTGS2 cells in primary neuron AD model. Comparisons of PTGS2 expression **(G,H)**, cell apoptosis **(I,J)**, and neurite outgrowth **(K,L)** among blank, NC, inhibitor-miR, Si-PTGS2, and inhibitor-miRandSi-PTGS2 cells in PC-12 cellular AD model. MiR-125b, microRNA-125b; PTGS2, prostaglandin-endoperoxide synthase 2; AD, Alzheimer disease; NC, negative control. *p < 0.05, **p < 0.01, ***p < 0.001.

**Figure 8:**
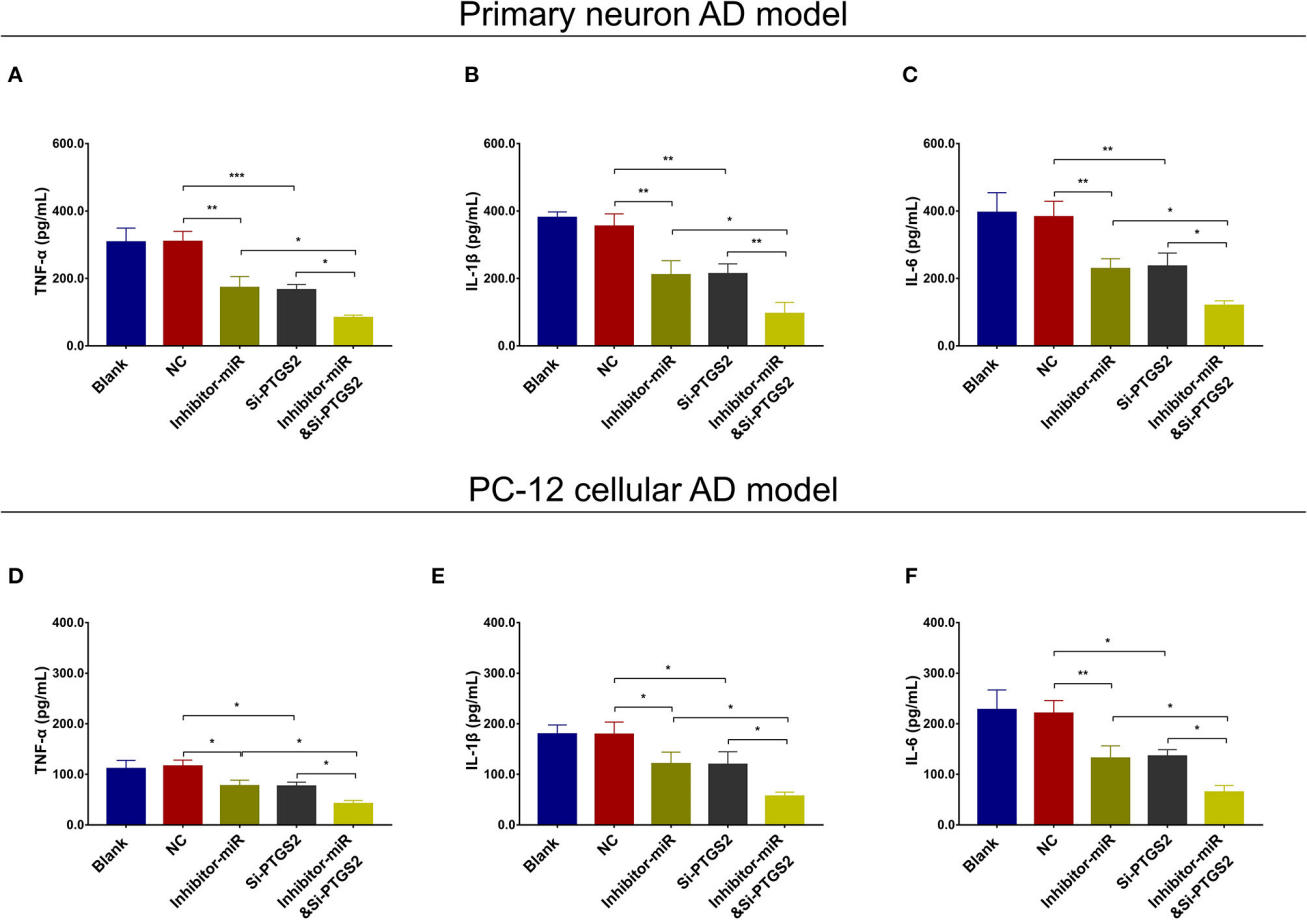
PTGS2 inhibition enhanced the effect of miR-125b inhibition on inflammation. Comparisons of TNF-α **(A)**, IL-1β **(B)**, and IL-6 **(C)** among blank, NC, inhibitor-miR, Si-PTGS2, and inhibitor-miRandSi-PTGS2 cells in primary neuron AD model. Comparisons of TNF-α **(D)**, IL-1β **(E)**, and IL-6 **(F)** among blank, NC, inhibitor-miR, Si-PTGS2, and inhibitor-miRandSi-PTGS2 cells in PC-12 cellular AD model. MiR-125b, microRNA-125b; PTGS2, prostaglandin-endoperoxide synthase 2; TNF-α, tumor necrosis factor-α; IL-1β, interleukin 1β; IL-6, interleukin 6; NC, negative control; AD, Alzheimer disease. *p < 0.05, **p < 0.01, ***p < 0.001.

In NC or miR-125b inhibitor–treated primary neuron AD model (Figures 7A–F), as well as in NC or miR-125b inhibitor–treated PC-12 cellular AD model (Figures 7G–L), Si-PTGS2 repressed PTGS2 expression and cell apoptosis but facilitated neurite outgrowth (Figures 7A–L); meanwhile, Si-PTGS2 also reduced TNF-α, IL-1β, and IL-6 levels (Figures 8A–F). Collectively, these findings indicated that miR-125b inhibition repressed cell apoptosis and inflammation but promoted neurite outgrowth via downregulating PTGS2 expression in AD.

### MiR-125b Inhibition Suppressed Cell Apoptosis by Downregulating CDK5

**Figure 9:**
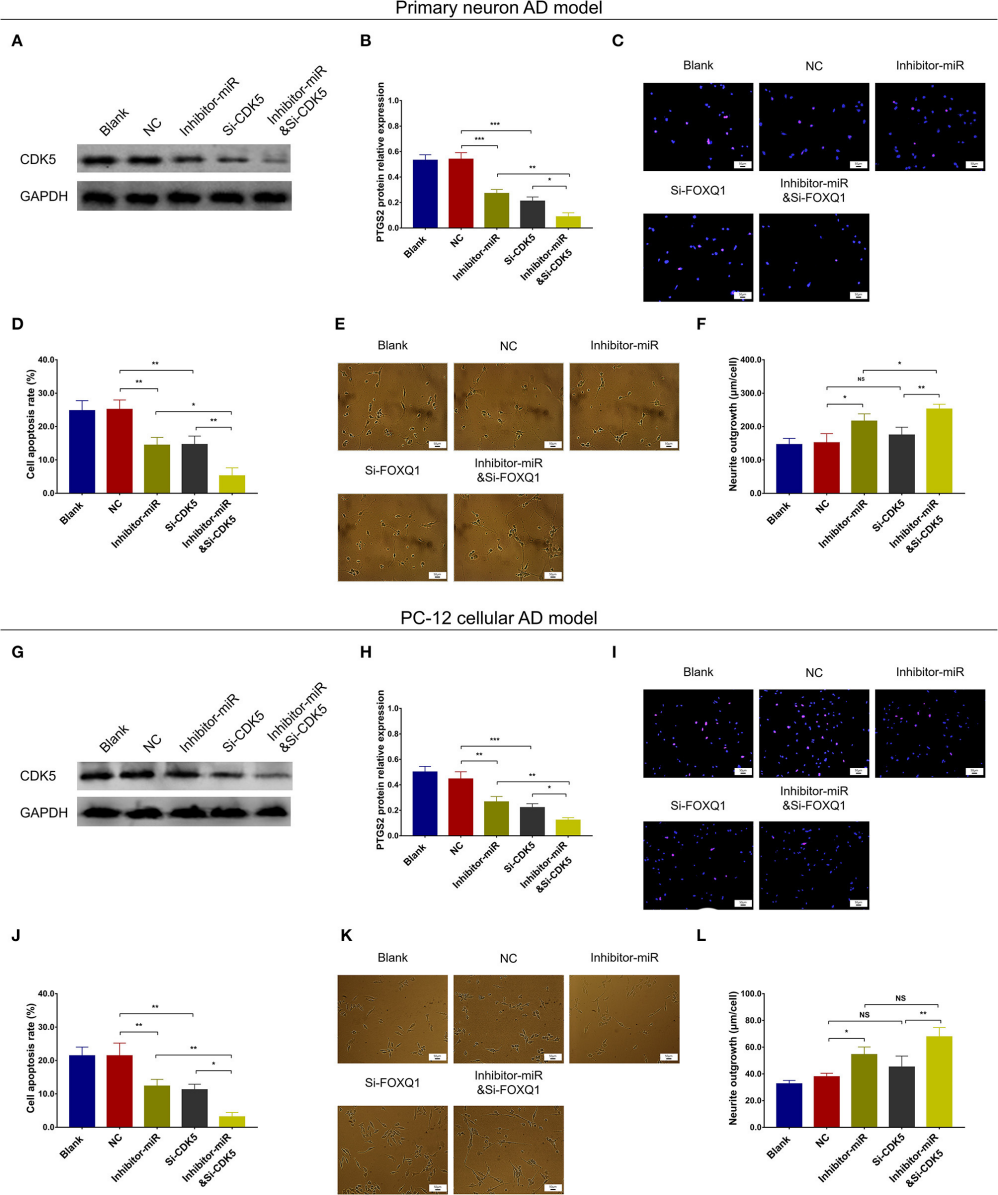
CDK5 inhibition enhanced the effect of miR-125b inhibition on cell apoptosis and neurite outgrowth. Comparisons of CDK5 expression **(A,B)**, cell apoptosis **(C,D)**, and neurite outgrowth **(E,F)** among blank, NC, inhibitor-miR, Si-CDK5, and inhibitor-miRandSi-CDK5 cells in primary neuron AD model. Comparisons of CDK5 expression **(G,H)**, cell apoptosis **(I,J)**, and neurite outgrowth **(K,L)** among blank, NC, inhibitor-miR, Si-CDK5, and inhibitor-miRandSi-CDK5 cells in PC-12 cellular AD model. MiR-125b, microRNA-125b; CDK5, cyclin-dependent kinase 5; NC, negative control; AD, Alzheimer disease. *p < 0.05, **p < 0.01, ***p < 0.001.

In both NC- and miR-125b inhibitor–treated primary neuron AD models, Si-CDK5 reduced CDK5 expression (Figures 9A,B) and cell apoptosis (Figures 9C,D). As to neurite outgrowth, Si-CDK5 enhanced neurite outgrowth in miR-125b inhibitor–treated primary neuron AD model but not in NC primary neuron AD model (Figures 9E,F). Furthermore, in both NC- and miR-125b inhibitor–treated PC-12 cellular AD models, Si-CDK5 attenuated CDK5 expression (Figures 9G,H) and cell apoptosis (Figures 9I,J), whereas it did not affect neurite outgrowth (Figures 9K,L).

**Figure 10:**
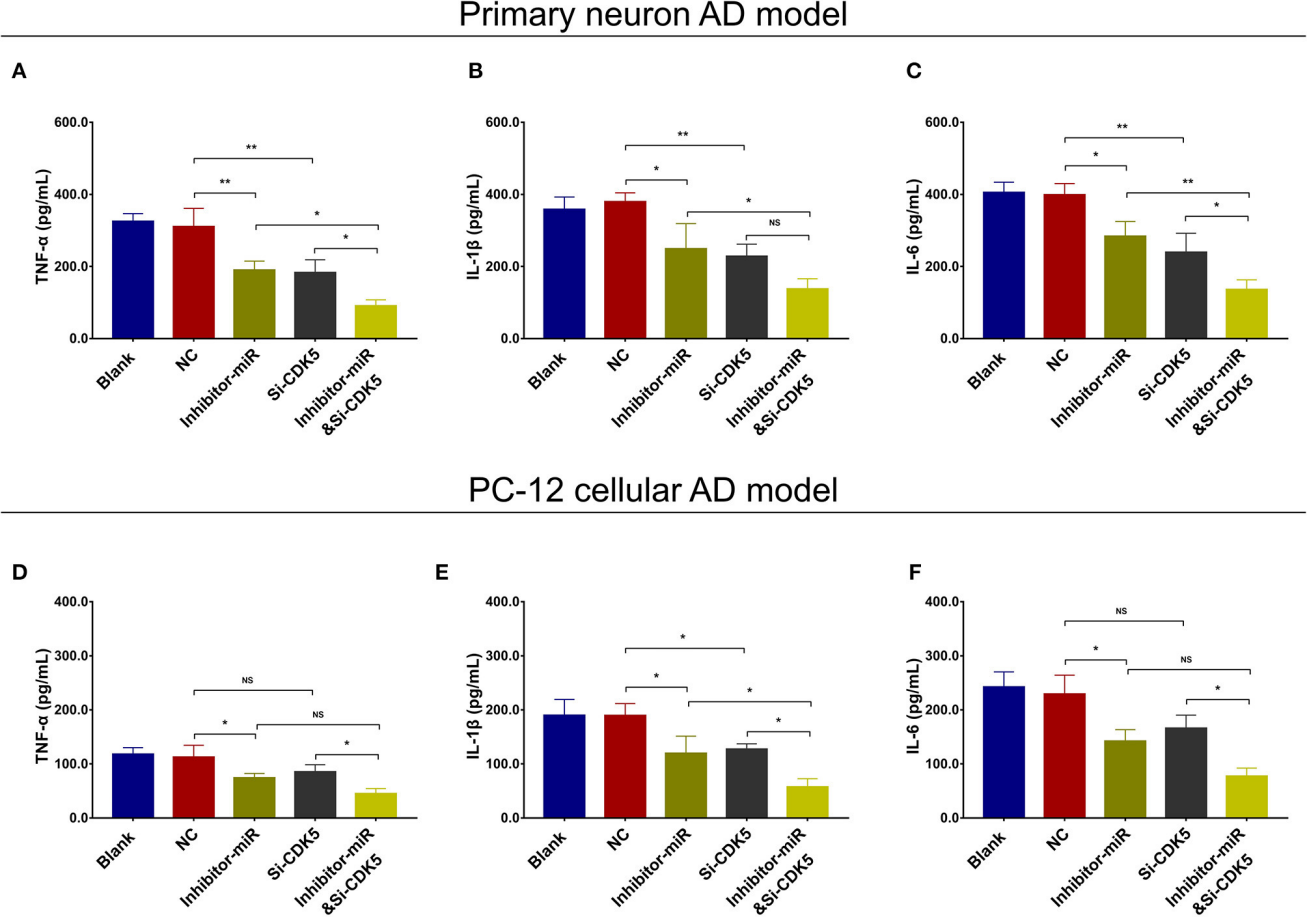
CDK5 inhibition enhanced the effect of miR-125b inhibition on inflammation. Comparisons of TNF-α **(A)**, IL-1β **(B)**, and IL-6 **(C)** levels among blank, NC, inhibitor-miR, Si-CDK5, and inhibitor-miRandSi-CDK5 cells in primary neuron AD model. Comparisons of TNF-α **(D)**, IL-1β **(E)**, and IL-6 levels **(F)** among blank, NC, inhibitor-miR, Si-CDK5, and inhibitor-miRandSi-CDK5 cells in PC-12 cellular AD model. MiR-125b, microRNA-125b; CDK5, cyclin-dependent kinase 5; TNF-α, tumor necrosis factor-α; IL-1β, interleukin 1β; IL-6, interleukin 6; NC, negative control; AD, Alzheimer disease. *p < 0.05, **p < 0.01, ***p < 0.001.

Regarding inflammation, Si-CDK5 inhibited TNF-α (Figure 10A), IL-1β (Figure 10B), and IL-6 (Figure 10C) levels in both NC- and miR-125b inhibitor–treated primary neuron AD models. Furthermore, Si-CDK5 attenuated IL-1β level (Figure 10E), whereas it did not affect TNF-α (Figure 10D) or IL-6 levels (Figure 10F) in NC and miR-125b inhibitor–treated PC-12 cellular AD models. Collectively, these findings indicated that miR-125b inhibition impeded cell apoptosis via downregulating CDK5 expression in AD, whereas the effect of miR-125b inhibition on neurite outgrowth and inflammation was less impacted by CDK5 in AD.

### FOXQ1 Inhibition Promoted Cell Apoptosis and Inflammation but Repressed Neurite Outgrowth by Enhancing PTGS2 and CDK5

**Figure 11:**
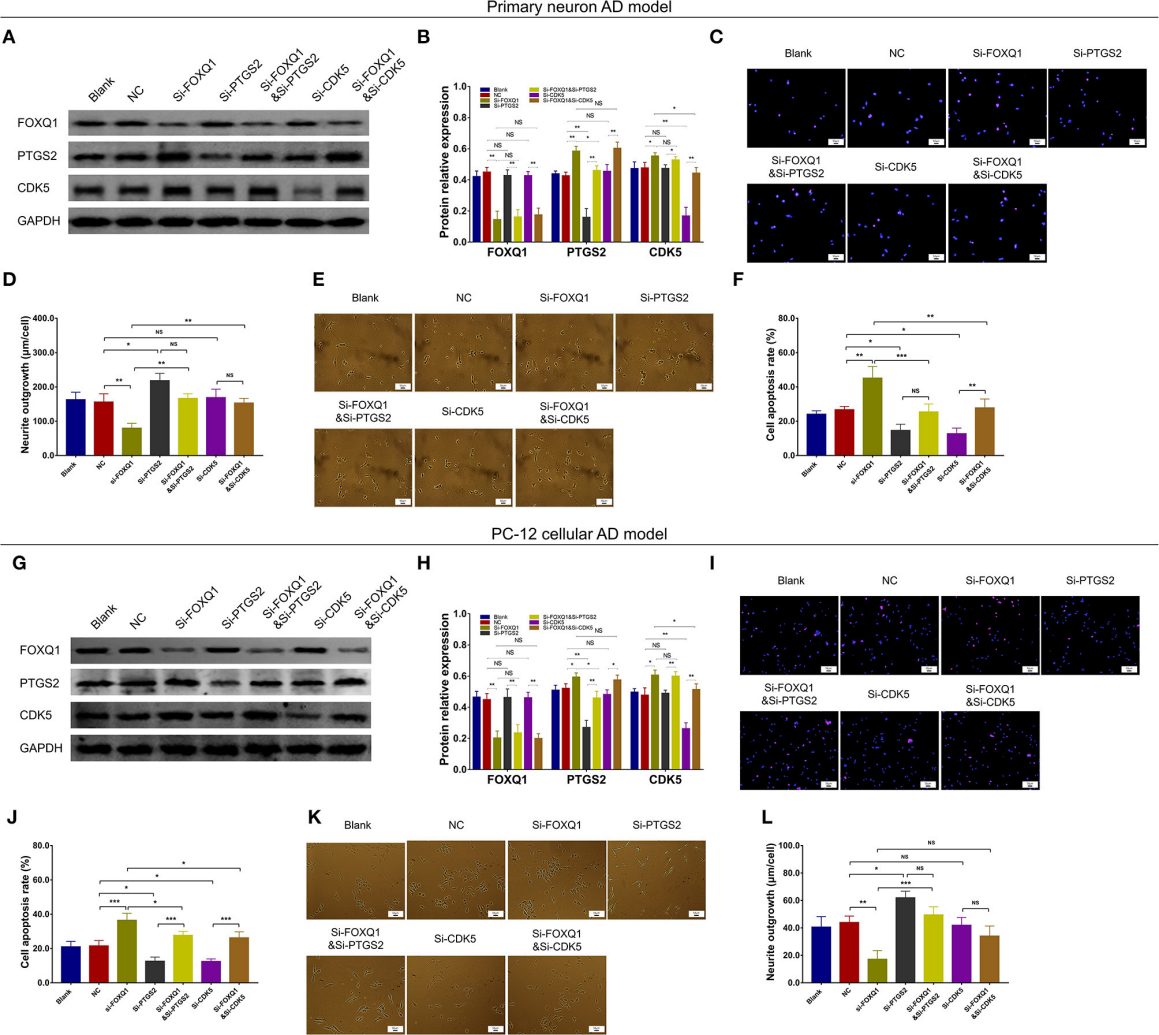
PTGS2 inhibition and CDK5 inhibition reversed the effect of FOXQ1 inhibition on cell apoptosis and neurite outgrowth. Comparisons of FOXQ1, PTGS2, CDK5 expressions **(A,B)**, cell apoptosis **(C,D)**, and neurite outgrowth **(E,F)** among blank, NC, Si-FOXQ1, Si-PTGS2, Si-FOXQ1andSi-PTGS2, Si-CDK5, and Si-FOXQ1andSi-CDK5 cells in primary neuron AD model. Comparisons of FOXQ1, PTGS2, CDK5 expressions **(G,H)**, cell apoptosis **(I,J)**, and neurite outgrowth **(K,L)** among blank, NC, Si-FOXQ1, Si-PTGS2, Si-FOXQ1andSi-PTGS2, Si-CDK5, and Si-FOXQ1andSi-CDK5 cells in PC-12 cellular AD model. FOXQ1, forkhead box Q1; PTGS2, prostaglandin-endoperoxide synthase 2; CDK5, cyclin-dependent kinase 5; NC, negative control; AD, Alzheimer disease. *p < 0.05, **p < 0.01, ***p < 0.001.

Based on above findings, miR-125b inhibition positively regulated FOXQ1 and negatively regulated PTGS2 and CDK5 to attenuate AD progression. While the inner regulation among FOXQ1, PTGS2, and CDK5 was still unknown, therefore, we further conducted FOXQ1 rescue experiments. In primary neuron AD model, PTGS and CDK5 expressions were obviously increased in Si-FOXQ1 cells compared with NC cells (Figures 11A,B). Meanwhile, in both NC- and FOXQ1 siRNA–treated primary neuron AD models, Si-PTGS2 reduced PTGS2 expression (Figures 11A,B) and cell apoptosis (Figures 11C,D) but promoted neurite outgrowth (Figures 11E,F). As for Si-CDK5, it attenuated CDK5 expression (Figures 11A,B) and cell apoptosis (Figures 11C,D) in both NC- and FOXQ1 siRNA–treated primary neuron AD models; meanwhile, Si-CDK5 enhanced neurite outgrowth in FOXQ1 siRNA–treated primary neuron AD model but not in NC primary neuron AD model (Figures 11E,F).

In PC-12 cellular AD model, PTGS2 and CDK5 expressions were obviously elevated in Si-FOXQ1 cells compared with NC cells (Figures 11G,H). Meanwhile, in both NC- and FOXQ1 siRNA–treated PC-12 cellular AD models, Si-PTGS2 reduced PTGS2 expression (Figures 11G,H) and cell apoptosis (Figures 11I,J) but elevated neurite outgrowth (Figures 11K,L). Regarding Si-CDK5, it attenuated CDK5 expression (Figures 11G,H) and cell apoptosis (Figures 11I,J), while Si-CDK5 did not affect neurite outgrowth (Figures 11K,L) in both NC- and FOXQ1 siRNA–treated primary neuron AD models.

**Figure 12:**
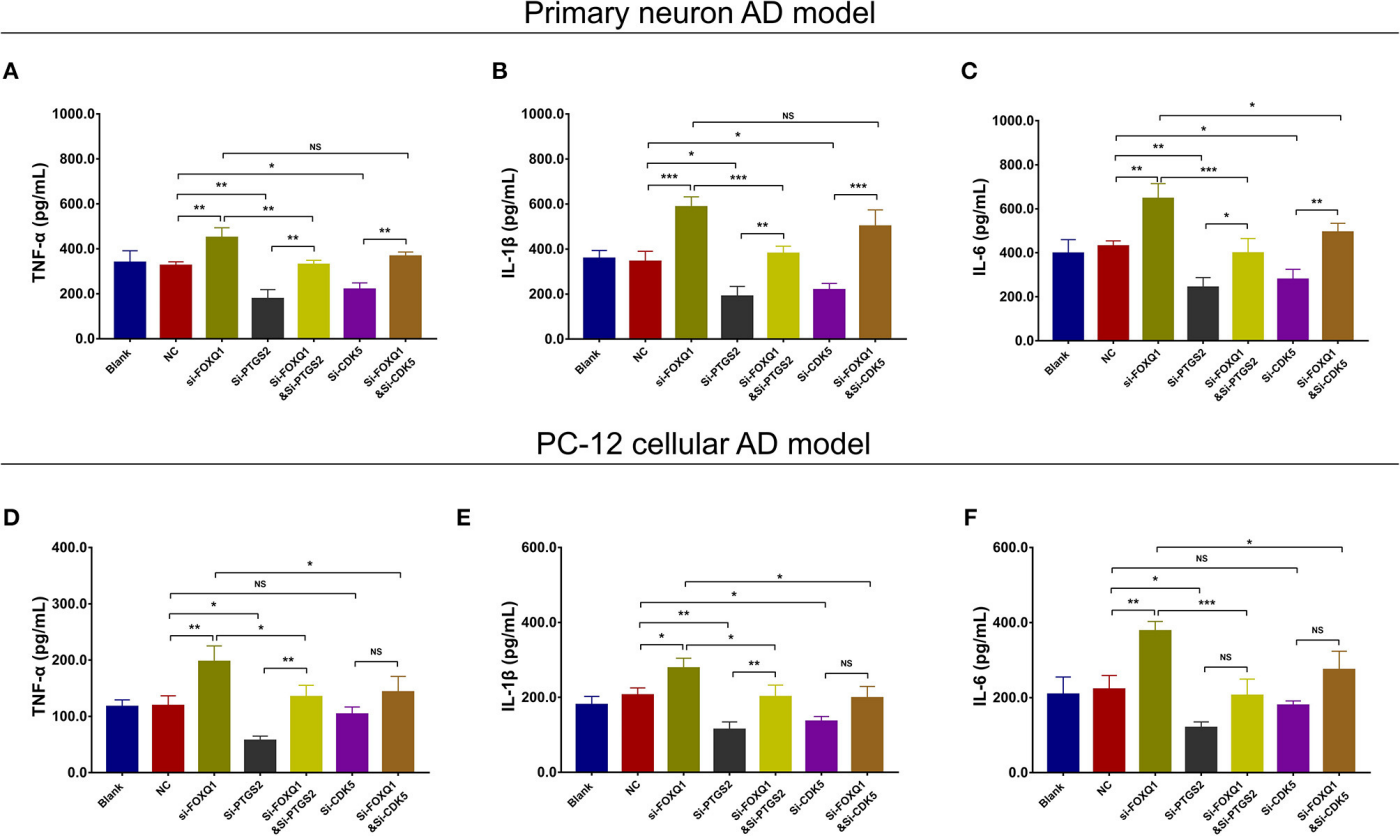
PTGS2 inhibition and CDK5 inhibition reversed the effect of FOXQ1 inhibition on inflammation. Comparisons of TNF-α **(A)**, IL-1β **(B)**, and IL-6 **(C)** levels among blank, NC, Si-FOXQ1, Si-PTGS2, Si-FOXQ1andSi-PTGS2, Si-CDK5, and Si-FOXQ1andSi-CDK5 cells in primary neuron AD model. Comparisons of TNF-α **(D)**, IL-1β **(E)**, and IL-6 **(F)** levels among blank, NC, Si-FOXQ1, Si-PTGS2, Si-FOXQ1andSi-PTGS2, Si-CDK5, and Si-FOXQ1andSi-CDK5 cells in PC-12 cellular AD model. FOXQ1, forkhead box Q1; PTGS2, prostaglandin-endoperoxide synthase 2; CDK5, cyclin-dependent kinase 5; TNF-α, tumor necrosis factor-α; IL-1β, interleukin 1β; IL-6, interleukin 6; NC, negative control; AD, Alzheimer disease. *p < 0.05, **p < 0.01, ***p < 0.001.

As to inflammation, in both NC- and FOXQ1 siRNA–treated primary neuron AD models, Si-PTGS2 repressed TNF-α (Figure 12A), IL-1β (Figure 12B), and IL-6 (Figure 12C) levels. Regarding Si-CDK5, it inhibited TNF-α (Figure 12A), IL-1β (Figure 12B), and IL-6 (Figure 12C) levels in NC primary neuron AD model, while Si-CDK5 only attenuated IL-6 level (Figure 12C) but not TNF-α (Figure 12A) or IL-1β (Figure 12B) level in FOXQ1 siRNA–treated primary neuron AD model.

In both NC- and FOXQ1 siRNA–treated PC-12 cellular AD models, Si-PTGS2 reduced TNF-α (Figure 12D), IL-1β (Figure 12E), and IL-6 (Figure 12F) levels. In terms of Si-CDK5, it only decreased IL-1β level (Figure 12E) but not TNF-α (Figure 12D) or IL-6 (Figure 12F) level in NC PC-12 cellular AD model; meanwhile, Si-CDK5 repressed all TNF-α (Figure 12D), IL-1β (Figure 12E), and IL-6 (Figure 12F) levels in FOXQ1 siRNA–treated PC-12 cellular AD model.

Collectively, these findings indicated that FOXQ1 inhibition facilitated cell apoptosis and inflammation but inhibited neurite outgrowth by enhancing PTGS2 and CDK5 expressions in AD. Combing with all the above findings, it was indicated that miR-125b inhibition suppressed cell apoptosis and inflammation but facilitated neurite outgrowth via downregulating PTGS2 and CDK5 in a FOXQ1-dependent way in AD.

### FOXQ1 Inhibition Upregulated PTGS2 and CDK5 but Not miR-125b in Primary Neurons and NGF-Stimulated PC-12 Cells

In primary neurons (Supplementary Figures 4A–C) and NGF-stimulated PC-12 cells (Supplementary Figures 4D–F), FOXQ1 expression was decreased, whereas PTGS2 and CDK5 expressions were increased in Si-FOXQ1 cells compared with NC cells; besides, miR-125b expression was of no difference between Si-FOXQ1 cells and NC cells.

### Molecular Network Among miR-125b, FOXQ1, PTGS2, and CDK5 on Regulating Cell Apoptosis, Neurite Outgrowth, and Inflammation

MiR-125b inhibition enhanced neurite outgrowth but suppressed cell apoptosis and inflammation via blocking PTGS2 and CDK5 in a FOXQ1-dependent way in AD cells (Supplementary Figure 5).

## Discussion

In the current study, we observed that in AD cellular models: (1) MiR-125b inhibition repressed cell apoptosis and inflammation but promoted neurite outgrowth; meanwhile, it elevated FOXQ1 but attenuated PTGS2 and CDK5. (2) The dysregulation of FOXQ1, PTGS2, and CDK5 compensated the effect of miR-125b inhibition on cell apoptosis, neurite outgrowth, and inflammation. (3) FOXQ1 inhibition enhanced cell apoptosis and inflammation but inhibited neurite outgrowth by enhancing PTGS2 and CDK5.

AD is a chronic disease with a long period of incubation before clinical symptoms emerge, and it is the leading cause of cognitive impairment in elderly people (Scheltens et al., 2016). Despite that the past 30 years of tremendous efforts in research on AD have proposed a variety of hypotheses regarding pathological and biochemical manifestations (including amyloid, tau, cholinergic, excitotoxicity, oxidative stress, ApoE, CREB signaling pathways, etc.), the development of effective treatments for halting the progression of AD symptoms or curing AD has been fruitless because of the complex and multifactorial pathophysiology (Graham et al., 2017). The aging global population and lack of effective treatments foreshadow a negative outlook. Hence, a more comprehensive understanding of underlying molecular mechanisms and pathophysiological pathways underlying AD may yield insights into the development of new, effective treatments of AD.

MicroRNAs, small noncoding RNAs (~18–25 nucleotides long), play a vital role in the regulation of gene expression at the posttranscriptional level (Guedes et al., 2013). As microRNAs are highly abundant in the brain, they are proposed as vital modulators for multiple brain functions in both physiological and pathological conditions (Godlewski et al., 2019). MiR-125b is one of the highly abundant microRNAs in the brain, and emerging evidence has implicated its involvement in multiple aspects of AD pathogenesis such as neuron apoptosis and tau phosphorylation (Cogswell et al., 2008; Banzhaf-Strathmann et al., 2014; Ma et al., 2017; Jin et al., 2018). As an example, miR-125b promotes the apoptosis of neurons and the phosphorylation of tau via activating p35/35 in the neuron cells in AD (Ma et al., 2017). Another study illuminates that miR-125b overexpression represses neuron proliferation but facilitates neuron apoptosis, inflammation, and oxidative stress via suppressed sphingosine kinase 1 in an *in vitro* model of AD (Jin et al., 2018). Additionally, in the study of our collaboration institutes, miR-125b overexpression enhances neuron apoptosis, inflammation, PTGS2, and CDK5 but represses neurite outgrowth and FOXQ1 in two *in vitro* models of AD (Ma et al., 2019). However, the molecular regulatory network of miR-125b, FOXQ1, PTGS2, and CDK5, as well as their effect on regulating the progression of AD cellular models, has not been elucidated yet.

In the current study, we initially evaluated the effect of Aβ_1−42_ treatment on FOXQ1, PTGS2, and CDK5. The results exhibited that in both primary neurons and NGF-stimulated PC-12 cells, FOXQ1 expression was reduced, whereas PTGS2 and CDK5 expressions were elevated in AD models (with Aβ_1−42_ treatment) compared with normal cells (without Aβ_1−42_ treatment), which indicated that Aβ_1−42_ treatment indeed decreased FOXQ1 expression but increased PTGS2 and CDK5 expressions. Then, we assessed the expression of miR-125b in primary neurons and NGF-stimulated PC-12 cells after Aβ_1−42_ insult, and we found that miR-125b was overexpressed in primary neurons and NGF-stimulated PC-12 cells. After that, the effect of miR-125b inhibition on cell apoptosis, neurite outgrowth, inflammation, and its target genes (FOXQ1, PTGS2, and CDK5) in AD was explored using AD cellular models. In both cellular AD models, we disclosed that miR-125b inhibition enhanced neurite outgrowth but impeded cell apoptosis and inflammation; miR-125b inhibition upregulated FOXQ1 but downregulated PTGS2 and CDK5. The possible reasons were that (i) MiR-125b probably enhanced the expression of downstream proteins such as p35 and CDK5 in the AD brain, which in turn induced subsequent tau hyperphosphorylation, the formation of NFTs, neuron dysfunction, and eventually neuron cell apoptosis; thereby, miR-125b inhibition decreased neuron cell apoptosis in AD (Dehghani et al., 2018; Jin et al., 2018); (ii) MiR-125b might inhibit the translation of downstream genes involved in the neurite outgrowth such as NCAM-140/180 translation, which subsequently decreased the expression of neural cell adhesion molecule that stimulated neurite outgrowth through the mediation of Ras–mitogen-activated protein kinase pathway; thereby, miR-125b inhibition promoted neurite outgrowth (Kolkova et al., 2000; Jessen et al., 2001; Zhang et al., 2019); (iii) MiR-125b might potentiate the activation of M1 microglia that induced the proinflammatory cascades by tau phosphorylation, which in turn exaggerated the NLRP3 inflammasome production and proinflammatory cytokine release; thereby, miR-125b inhibition repressed inflammation in AD models (Chaudhuri et al., 2011; Zhang et al., 2017; Dehghani et al., 2018).

Furthermore, in this current study, we performed miR-125b rescue experiments in two AD cellular models. We discovered that FOXQ1 inhibition promoted cell apoptosis and inflammation but repressed neurite outgrowth; PTGS2 inhibition suppressed cell apoptosis and inflammation but enhanced neurite outgrowth; CDK5 inhibition attenuated cell apoptosis, while it only facilitated neurite outgrowth and repressed inflammation to some extent. Additionally, FOXQ1 inhibition reversed the effect of miR-125 inhibition on cell apoptosis, inflammation, and neurite outgrowth, whereas PTGS2 inhibition and CDK5 inhibition achieved opposite effects. These findings implied that miR-125b inhibition suppressed cell apoptosis and inflammation but facilitated neurite outgrowth via upregulating FOXQ1 but downregulating PTGS2 and CDK5 in AD. Herein, several possible explanations were proposed: (i) FOXQ1 might transactivate gene expression of p21 (G1 cyclin kinase inhibitor), which arrested cells in G_0_/G_1_ phase, promoted neurite outgrowth, and repressed cell apoptosis in AD; thereby, FOXQ1 inhibition enhanced cell apoptosis and inflammation but repressed neurite outgrowth (Park et al., 2012). (ii) PTGS2, a key enzyme involved in inflammation, induced the production of IL-1β and Aβ through the activation of the PI3-K/AKT and PKA/CREB pathways in neurons, which facilitated cell apoptosis and inflammation and reduced neurite outgrowth; thereby, PTGS2 inhibition suppressed cell apoptosis and inflammation but facilitated neurite outgrowth (Yang et al., 2018). (iii) CDK5 promoted the aberrant hyperphosphorylation amyloid precursor protein, tau, and neurofilament, which contributed to the formation of neurofibrillary tangles, synaptic damage, mitochondria dysfunction to cell cycle reactivation, and neuronal cell apoptosis in AD (Patrick et al., 1999; Liu et al., 2016); besides, CDK5 hyperactivation might trigger glia to produce proinflammatory cytokines and chemokines by regulating cPLA2, which contributed to neuroinflammation; thereby, CDK5 inhibition repressed cell apoptosis and inflammation in AD (Sundaram et al., 2012). (iv) Based on the aforementioned evidences, through upregulating FOXQ1 but downregulating PTGS2 and CDK5, miR-125b inhibition suppressed cell apoptosis and inflammation but facilitated neurite outgrowth in AD. Interestedly, the effect of miR-125b inhibition on neurite outgrowth and inflammation was less impacted by CDK5 inhibition in AD. This was likely to be explained by miR-125b inhibition itself and greatly promoted neurite outgrowth and repressed inflammation, and CDK5 inhibition could only enhance neurite outgrowth and inhibited inflammation to some extent; thus, the effect of CDK5 inhibition on modulating miR-125b inhibition–mediated neurite outgrowth and inhibited inflammation was not obvious in AD.

In addition, the inner regulation among FOXQ1, PTGS2, and CDK5 on regulating cell activities in AD was still unknown; we further performed FOXQ1 rescue experiments in two AD cellular models. We found that FOXQ1 inhibition enhanced cell apoptosis and inflammation but suppressed neurite outgrowth by enhancing PTGS2 and CDK5 in AD. Herein, this finding could be explained by the following: PTGS2 probably amplified the production of inflammatory chemokines and cytokines via activating its downstream pathways such as PI3-K/AKT and PKA/CREB pathways, which in turn enhanced neuron apoptosis and inflammation and repressed neurite outgrowth (Yang et al., 2018). Furthermore, CDK5 might intensify the release of proinflammatory cytokines and chemokines in glia by modulating its downstream pathway such as cPLA2, which induced neuroinflammation and neuron apoptosis (Patrick et al., 1999; Sundaram et al., 2012; Liu et al., 2016). Additionally, FOXQ1 might activate the transcription of PTGS2 and CDK5 directly by binding to the promoters of PTGS2 and CDK5, while further experiments were needed for validating our speculation. These data indicated that FOXQ1 inhibition downregulated PTGS2 and CDK5 to facilitate cell apoptosis and inflammation but repressed neurite outgrowth in AD. Of note, owing to the limited budget, only AD cellular models were included for the explorations; thus, further animal study would be desirable to validate our results.

To conclude, miR-125b inhibition promotes neurite outgrowth but represses cell apoptosis and inflammation via blocking PTGS2 and CDK5 in a FOXQ1-dependent way in AD, which might provide the basis for developing potential drug targets for AD treatment.

## Data Availability Statement

The original contributions presented in the study are included in the article/Supplementary Materials, further inquiries can be directed to the corresponding author/s.

## Ethics Statement

The animal study was completed in accordance with the National Guideline for Experimental Animal Welfare and approved by the Animal Ethics Committee of our institution.

## Author Contributions

JZ conceived of the study. ZC, PC, and RW designed the data analysis, with QY and LL conducting the data analysis. HY and RZ wrote the first draft of the original protocol. All authors revised the manuscript and approved the final version of the article. JZ is the article guarantor.

## Conflict of Interest

The authors declare that the research was conducted in the absence of any commercial or financial relationships that could be construed as a potential conflict of interest.
