# Supplementary material for: Targeting MicroRNA-125b Promotes Neurite Outgrowth but Represses Cell Apoptosis and Inflammation via Blocking PTGS2 and CDK5 in a FOXQ1-Dependent Way in Alzheimer Disease
Source: Front Cell Neurosci. 2020 Dec 21;14:587747. doi: 10.3389/fncel.2020.587747 (PMC7779636; doi:10.3389/fncel.2020.587747)
Supplement: Supplementary file 1 [file Data_Sheet_1.pdf]

Supplementary Material

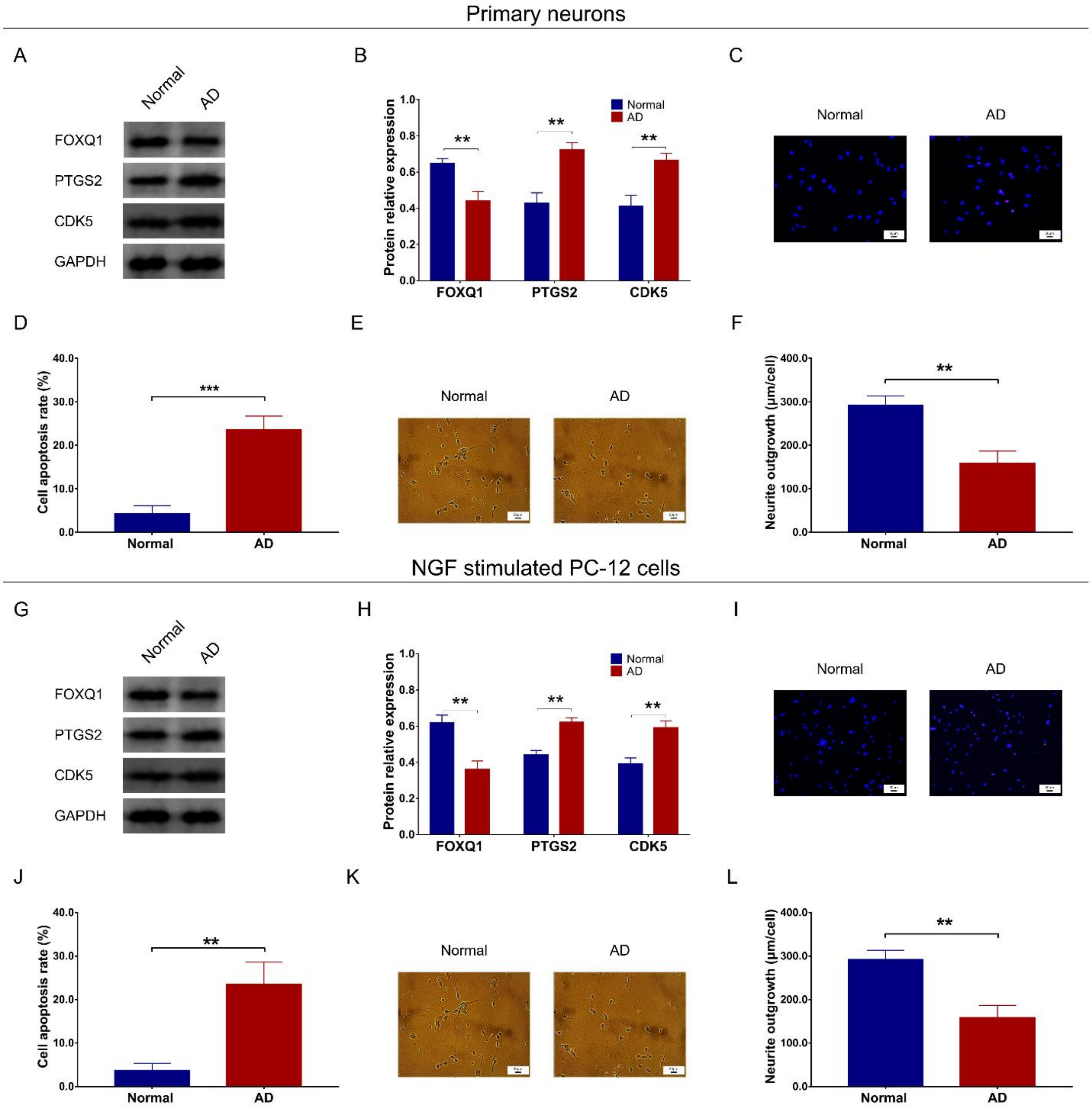

Supplementary figure 1. Effect of Aβ_{1-42} treatment on FOXQ1, PTGS2, CDK5, cell apoptosis and neurite outgrowth. Comparisons of FOXQ1, PTGS2, CDK5 protein expressions (**A, B**), cell apoptosis (**C, D**) and neurite outgrowth (**E, F**) between normal cells and AD cells in primary neurons. Comparisons of FOXQ1, PTGS2, CDK5 protein expressions (**G, H**), cell apoptosis (**I, J**) and neurite outgrowth (**K, L)** between normal cells and AD cells in NGF stimulated PC-12 cells. FOXQ1, Forkhead box Q1; PTGS2, Prostaglandin-endoperoxide synthase 2; CDK5, Cyclin-dependent Kinase 5; AD, Alzheimer's Disease; NGF, nerve growth factor.

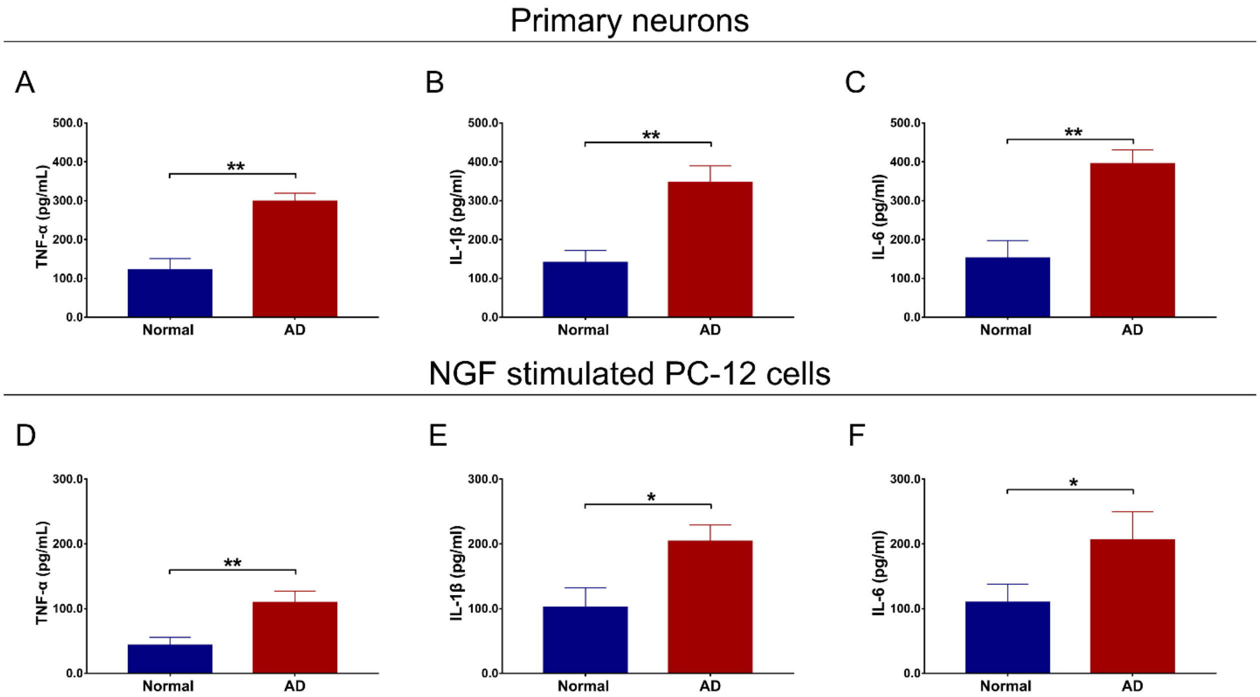

Supplementary figure 2. Effect of Aβ_{1-42} treatment on inflammation. Comparisons of TNF-α (**A**), IL-1β(**B**) and IL-6 (**C**) levels between normal cells and AD cells in primary neurons. Comparisons of TNF-α (**D**), IL-1β (**E**) and IL-6 (**F**) levels between normal cells and AD cells in NGF stimulated PC-12 cells. TNF-α,tumornecrosisfactor-α;IL-1β,interleukin-1β;IL-6, interleukin-6; AD, Alzheimer's Disease; NGF, nerve growth factor.

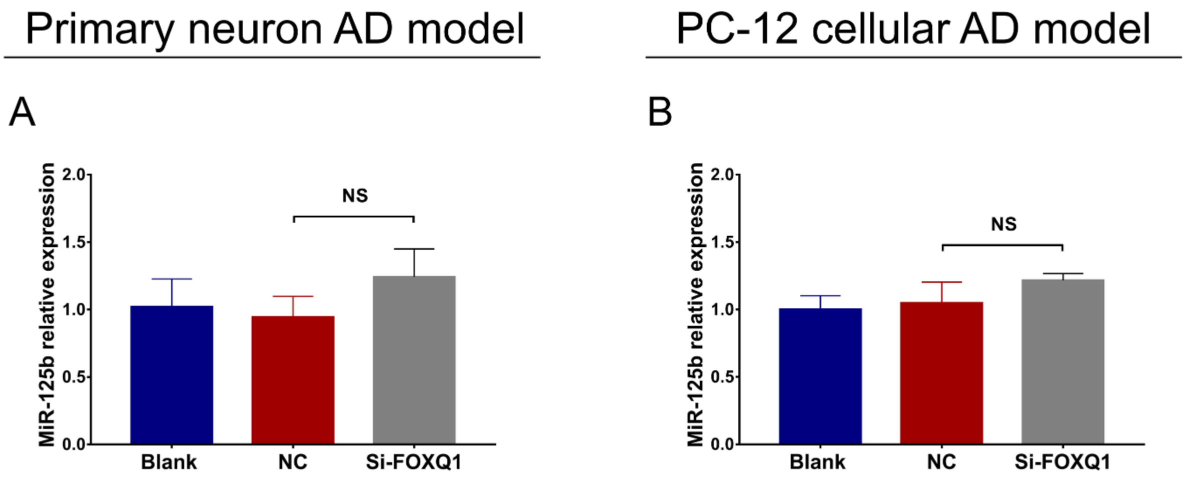

Supplementary figure 3. Effect of FOXQ1 inhibition on miR-125b expression in AD cellular models. Comparison of miR-125b expression among blank, NC and Si-FOXQ1 cells in primary neuron AD model (**A**). Comparison of miR-125b expression among blank, NC and Si-FOXQ1 cells in PC-12 cellular AD model (**B**). FOXQ1, Forkhead box Q1; miR-125b, microRNA-125b; NC, negative control; AD, Alzheimer's Disease.

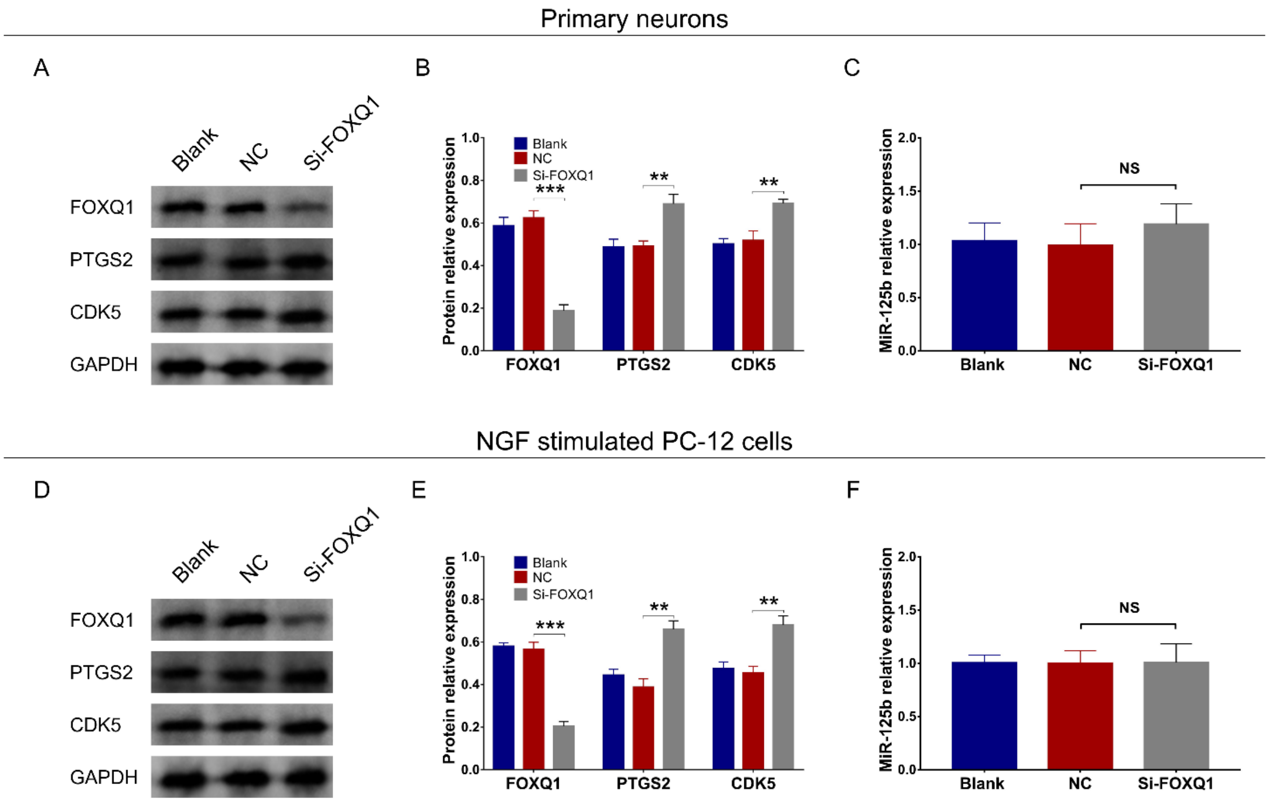

Supplementary figure 4. Effect of FOXQ1 inhibition on PTGS2, CDK5 and miR-125b expressions in primary neurons and NGF stimulated PC-12 cells. Comparisons of PTGS2, CDK5 protein expressions (**A, B**) and miR-125b expression (**C**) among blank, NC and Si-FOXQ1 cells in primary neurons. Comparisons of PTGS2 and CDK5 protein expressions (**D, E**) and miR-125b expression (**F**) among blank, NC and Si-FOXQ1 cells in NGF stimulated PC-12 cells. FOXQ1, Forkhead box Q1; PTGS2, Prostaglandin-endoperoxide synthase 2; CDK5, Cyclin-dependent Kinase 5; AD, Alzheimer's Disease; NC, negative control; NGF, nerve growth factor.

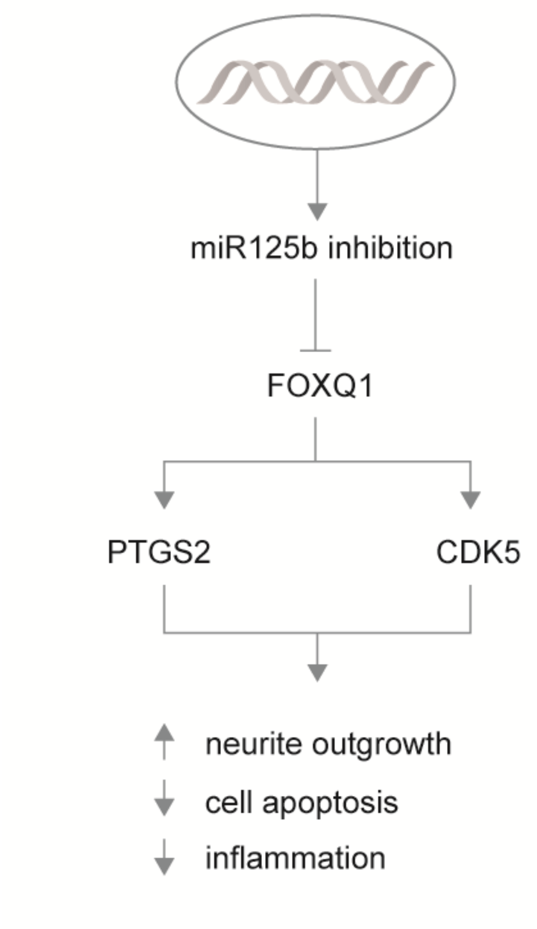

Supplementary figure 5. Cartoon depicting the molecular network among miR-125b, FOXQ1, PTGS2 and CDK5 on regulating cell apoptosis, neurite outgrowth and inflammation in AD. MiR-125b, microRNA-125b; FOXQ1, Forkhead box Q1; PTGS2, Prostaglandin-endoperoxide synthase 2; CDK5, Cyclin-dependent Kinase 5; AD, Alzheimer's Disease.
